# Supplementary material for: An Analysis of Children Left Unattended in Parked Motor Vehicles in Brazil
Source: Int J Environ Res Public Health. 2016 Jul 7;13(7):649. doi: 10.3390/ijerph13070649 (PMC4962190; doi:10.3390/ijerph13070649)
Supplement: Supplementary file 1 [file ijerph-13-00649-s001.pdf]

Supplementary Materials: An Analysis of Children Left Unattended in Parked Motor Vehicles in Brazil

Driely Costa and Andrew Grundstein

Table S1. Dataset of children left unattended in parked motor vehicles in Brazil, 2006–2015.

Case	Year	Month	Day	City	State	Age (Months)	Gender	*T*max (°C)	Circumstance	Fatal	Daycare	Responsible Adult
1	2006	4	12	São Paulo	SP	15	Male	27	Forgotten	Yes	Yes	Father
2	2007	4	12	Guarulhos	SP	16	Male	24	Forgotten	Yes	No	Father
3	2007	4	13	Altamira	PA	24	Male	31	Gained access	Yes	No	Grandparent
4	2008	1	5	Porto Alegre	RS	24	Male	29.4	Forgotten	Yes	No	Father
5	2008	6	8	Demerval	PI	48	Male	32.2	Left Behind	No	No	Father
6	2009	11	18	São Paulo	SP	6	Female	32	Forgotten	Yes	No	Mother
7	2009	11	18	São Paulo	SP	5	Female	32.2	Forgotten	Yes	No	Mother
8	2009	11	26	São Paulo	SP	6	Female	30	Forgotten	Yes	Yes	Mother
9	2009	12	3	Jundiaí	SP	18	Female	28.9	Left Behind	No	No	Mother
10	2010	7	27	São Paulo	SP	No info	Male	16.1	Left Behind	No	No	Mother
11	2011	5	6	Novo Hamburgo	RS	7	Female	24	Left Behind	Yes	Yes	Father
12	2011	12	12	Volta Redonda	RJ	4	Female	25.6	No info	No	No	Parents
13	2012	3	27	Aparecida de Goiania	GO	12	Male	32	Left Behind	Yes	No	Mother
14	2012	8	10	Registro	SP	12	Female	26.1	Forgotten	No	No	Mother
15	2012	11	9	Volta Redonda	RJ	10	Female	28	Forgotten	Yes	Yes	Father
16	2013	1	18	Santa Rosa	RS	11	Female	31.3	Forgotten	Yes	Yes	Father
17	2013	2	14	Divinópolis	MG	7	Male	33	Forgotten	Yes	Yes	Father
18	2013	3	14	Divinópolis	MG	36	Female	30	Forgotten	No	Yes	Male Childcare
19	2013	6	19	Lucas do Rio Verde	T	36	Female	32.8	Forgotten	Yes	Yes	Female Childcare
20	2013	11	14	Americana	SP	23	Male	30	Forgotten	Yes	Yes	Father
21	2013	11	22	Campo Grande	MS	15	Female	28.3	Forgotten	Yes	No	Father
22	2013	12	13	Cuiaba	MT	24	Male	32	Forgotten	Yes	Yes	Father
23	2014	9	15	Machado	MG	48	Male	36.7	Forgotten	No	Yes	Male Childcare
24	2014	10	27	Taubaté	SP	7	No info	30.6	Left Behind	No	No	Father
25	2014	12	13	Rio de janeiro	RJ	24	Male	29	Forgotten	Yes	Yes	Female Childcare
26	2014	12	17	São Bernardo do Campo	SP	28	Male	29	Forgotten	Yes	Yes	Father
27	2014	12	18	Belo Horizonte	MG	23	Female	29	Forgotten	Yes	Yes	Mother
28	2015	1	1	Curitiba	PR	6	Female	28.3	Forgotten	Yes	No	Mother
29	2015	2	6	Santos	SP	72	Male	25	Forgotten	Yes	Yes	Aunt
30	2015	4	5	Pouso Alegre	MG	83	Male	25.6	Left Behind	No	No	Mother
30	2015	4	5	Pouso Alegre	MG	120	Female	25.6	Left Behind	No	No	Mother
31	2015	8	3	Ilheus	BA	60	Male	27.2	Forgotten	No	Yes	Female Childcare

© 2016 by the authors; licensee MDPI, Basel, Switzerland. This article is an open access article distributed under the terms and conditions of the Creative Commons by Attribution (CC-BY) license (http://creativecommons.org/licenses/by/4.0/).
